# Evaluation of the occlusal trueness among 3-Dimensionally printed CAD-CAM complete dentures with different socket designs and teeth splinting assemblies

**DOI:** 10.1186/s12903-025-05894-7

**Published:** 2025-04-24

**Authors:** Rowan Mohamed Abdelsadek Mohamed Sallam, Nayrouz Adel Metwally, Mohamed Moataz Khamis

**Affiliations:** 1 Department of Prosthodontics, Faculty of Dentistry, Alexandria University, Alexandria, Egypt; 2 Department of Prosthodontics, Faculty of Dentistry, Alexandria University, Champollion St, Alexandria, Azarita Egypt

**Keywords:** 3D Printing, Accuracy, Complete dentures, CAD-CAM

## Abstract

**Background:**

Teeth sockets of the 3 dimensionally (3D) printed denture base can be designed in different shapes. Denture teeth can also be printed separately or splinted. However, the effect of the socket forms and the denture teeth splinting on the teeth displacement has not been clarified in literature. The goal of this study is to evaluate and compare the effect of different socket designs and teeth splinting assemblies on the trueness of the teeth positions of 3D-printed complete dentures.

**Methods:**

A total of 80 maxillary dentures were fabricated for this study. All dentures were designed by using a computer-aided design (CAD) software program (exocad software; exocad GmbH). Two designs for the teeth sockets of the denture base were used in this study: socketed design and thimble design. Teeth were also aligned in 3 forms: One-unit splinted design, 3-unit splinted design or unsplinted denture teeth. The dentures were divided into 8 groups (*n* = 10) according to the design used. Group I (unsplinted teeth/socketed base), Group II (unsplinted teeth/thimbled base), Group III (1-unit splinted teeth/socketed base), Group IV (1-unit splinted teeth/thimble base), Group V (3-unit splinted teeth/socketed base), Group VI (3-unit splinted teeth/thimble base), Group VII (Semi conventional pack and press), Group VIII (Monoblock). Groups from I -VII were 3D-printed by using SLA 3D-printer (Form 2; Formlabs Inc.) (Denture teeth A2, Formlabs) (Denture base LP, Formlabs). Group VIII was 3D-printed from castable wax resin (Castable wax, Formlabs Inc.) then flasked in a conventional manner. All dentures were then scanned by using a desktop scanner (Medit T710, Medit Corp) and saved as STL files. To evaluate the accuracy of the teeth position the CAD design file was imported and set as the reference data to which all scanned dentures were matched and compared by using (Geomagic Control X; 3D system Inc) The Shapiro–Wilk test of normality was used. Data was not normally distributed. Comparison between the study groups was done by using Krauskal Wallis test. Significance level was set at (*P* <.05).

**Results:**

The results showed a significant difference in teeth deviation values among the groups (*P* <.05). The lowest deviation values were reported in group VII across the overall denture teeth (0.104), anterior denture teeth (0.104) and posterior denture teeth (0.104), regarding the overall denture teeth and the posterior denture teeth, a statistically significant difference was identified when group VII was compared to all other groups. Regarding the anterior denture teeth, a non-statistically significant difference was identified when the group VII is compared to the group III. Higher deviation values were identified in the incisors as compared to the canines. However, deviation values were variable when the premolars and molars were compared.

**Conclusions:**

The results of the present study reported that median deviations were in the range of (0.104–0.282) mm, recommending the clinical choice of group VII followed by group III to provide the highest occlusal trueness.

## Background

CAD CAM dentures are commonly utilized worldwide as they have been reported to provide adequate patient satisfaction and reduce the number of clinical visits required, thereby reducing material wastage [1, 2]. However, problems with complete denture occlusion have been reported in conventional techniques as well as digital techniques of complete denture fabrication [3]. Occlusal inaccuracies consequently extend the chair side time required for occlusal adjustments, they poorly affect denture retention and stability and, they could substantially alter the occlusal anatomy, thereafter, influencing the occlusal scheme fabricated [4]. It has been reported that minimal denture tooth movement of 0.25 mm in an occlusal direction, can result in 1 mm incisal pin opening which subsequently alters the vertical dimension [5].

Goodacre et al. have reported that milled monoblock dentures provide the highest occlusal accuracy. However, monoblock dentures fabricated from regular bi-coloured Polymethylmethacrylate (PMMA) discs would either require extensive laboratory handling to produce accurate pink and white portions of the gingival margin and teeth [6] or the pink and white esthetics would be compromised. A Customized bi-coloured disc with shell geometry was developed allowing fabrication of accurate gingival margins. In addition to, providing optimum occlusion while eliminating any laboratory handling [7]. However, these discs are expensive and require special software modules [6, 8, 9]. A more commonly used approach is to bond the denture teeth to the denture base [10, 11]. This technique is employed in additive manufacturing as well as subtractive manufacturing. Despite that this method provides optimum pink and white aesthetics yet, the occlusion is compromised [8, 12]. Milled dentures produced as 2 separate units could be re-inserted after bonding in the milling machine to finalize the contours, thereby reducing the occlusal errors caused by bonding [7]. Conversely, dentures fabricated by 3D printing cannot be remodified by the computer aided manufacturing (CAM) process after fabrication. Thus, achieving the most accurate occlusion in 3D printed dentures is essential.

Dentures fabricated using subtractive manufacturing have superior mechanical properties [13] yet, in some parts of the world the laboratory costs remain unaffordable in comparison to heat cure dentures as well as 3D printed dentures [10]. Moreover, limitations to cases with extensive undercuts still exist even with 5 axis milling machines [14]. 3D Printing presents a financially efficient solution as multiple dentures could be printed simultaneously, less waste material is produced in comparison to milling [15]. And, the accuracy of 3D-printed dentures is not limited by the size of the milling burs [14, 16, 17].

Limitations of 3D printed dentures include poor bond strength between the denture base and the denture teeth as well as poor mechanical properties [18, 19]. Basal tooth forms as thimbles could provide a macro-mechanical means of retention to enhance the bond strength between the denture base and the denture teeth [20]. However, the effect on the occlusal accuracy remains unclear. Moreover, cases where high mechanical properties are required, yet the cost of milled dentures is unaffordable, a combined technique that comprises conventional and digital methods could be employed.

Thabet et al. [12] have investigated the effect of a guide on the occlusal accuracy of 3D printed dentures fabricated with separate and full arch teeth groups and compared that to monoblock 3D printed dentures. Deng et al. [6] have reported a technique where the denture is 3D printed using castable wax resin and is used as a wax pattern in which the denture teeth are set and the heat cure acrylic resin is packed in a conventional method. This method provides a cost-effective approach without compromising the mechanical properties. Up to the authors knowledge, studies that compare the occlusal trueness of this method to other forms of computer aided manufacturing are lacking. Moreover, studies evaluating occlusal trueness have not investigated 3-unit denture teeth assemblies, neither thimble basal tooth forms nor compared that to the combined conventional/digital method, making it difficult for the dentist and the dental technician to decide which denture teeth assembly, basal socket form or a semi-conventional pack and press technique would yield the highest occlusal trueness. According to the ISO 5725–1:2023 [21]"trueness"and"precision"describe the accuracy of a measurement method."Trueness"refers to the closeness of agreement between the arithmetic mean of a large number of test results and the true or accepted reference value."Precision"refers to the closeness of agreement between test results obtained under stipulated conditions. This paper investigates the effect of incorporating thimbles as basal tooth forms that might enhance the retention, by providing a macro-mechanical means of retention between the denture base and the denture teeth on the occlusal trueness. Furthermore, the effect of splinting denture teeth on the occlusal trueness is investigated and compared to semi-conventional pack and press dentures where a 3D printed denture castable resin is used as a wax pattern in which the denture teeth are set, the null hypothesis was that no significant difference would be found between the studied groups.

## Methods

### Sample size calculation

Six different methods for attaching 3D-printed denture teeth to 3D-printed denture bases were evaluated and compared to a monolithic SLA printed denture group as well as a semi-conventional pack and press denture group. Ten maxillary dentures were fabricated for each group (*n*= 80). Sample size was estimated assuming 5% alpha error and 80% study power. The mean ± SD tooth movement in occlusal direction for the conventional denture was 0.20 ± 0.11 mm and − 0.04 ± 0.09 mm for the CAD-CAM socket-shaped denture [8]. To compare these to two groups where estimates were available, 8 specimens are required per group. This was increased to 10 specimens to make up for processing errors. Total sample size = number per group × number of groups = 10 × 8 = 80 specimens. Sample size was based on Rosner’s method and was calculated by Gpower 3.0.10.

### Data acquisition

Edentulous maxillary and mandibular stone models (Elite Rock Dental Stone, Zhermack) that correspond to type A American College of Prosthodontists classification of residual ridge morphology were obtained from a rubber mold (EDE1001-UL-MO; Nissin Dental Products Inc) [22]. These reference models were scanned by using a desktop scanner providing a reference scan (Medit T710, Medit software version 1.2.7) [23]. Record blocks were fabricated and scanned by using the same desktop scanner. The scanned data were exported as standard tessellation language (STL) file for denture design.

### CAD design

**Fig. 1:**
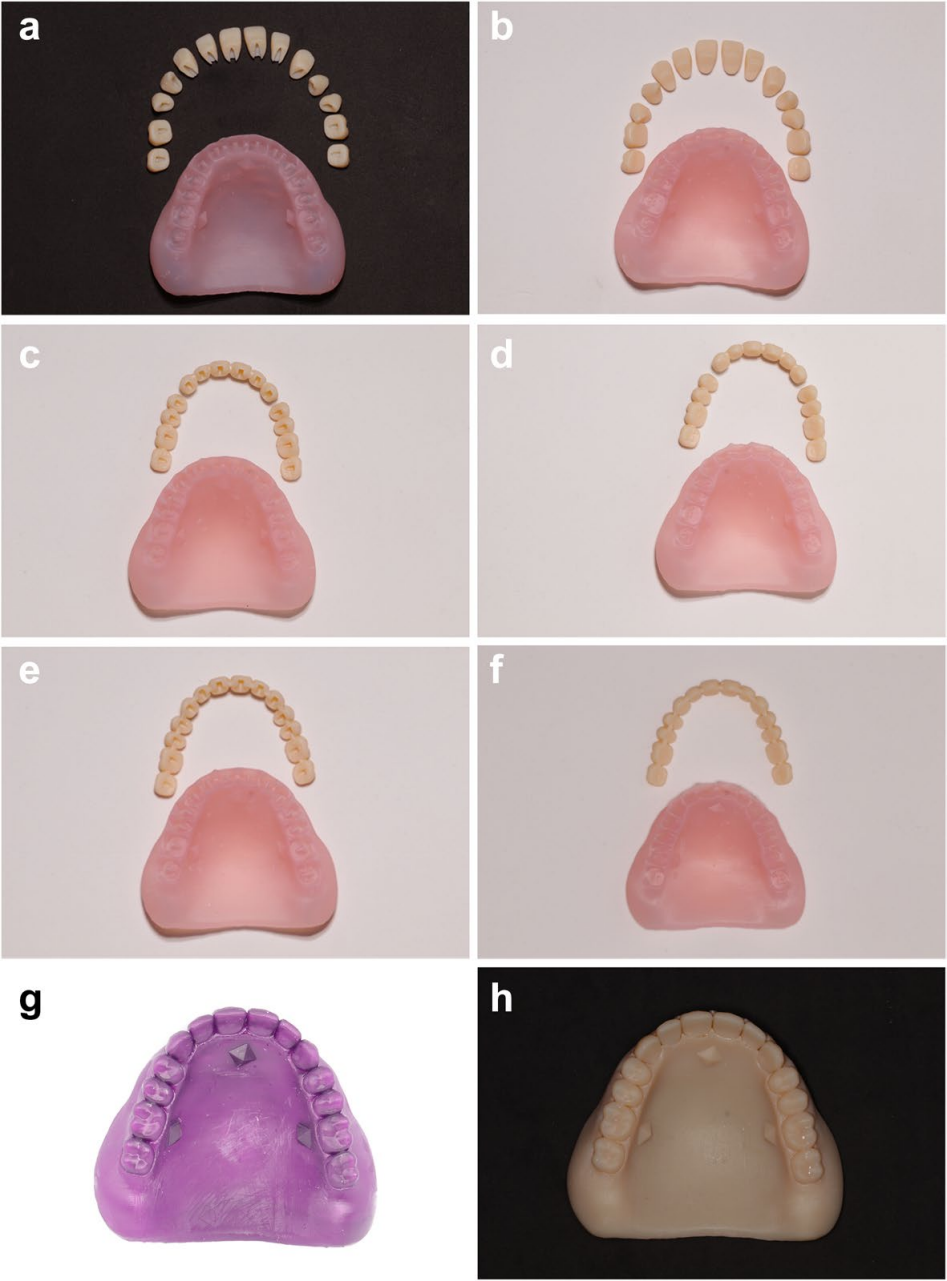
Different denture design groups. **A** Unsplinted socketed denture. **B** Unsplinted thimble denture. **C** 1-unit splinted socketed denture. **D** 1-unit thimbled denture. **E** 3-unit splinted socketed denture. **F** one-unit splinted thimble denture. **G **3D-printed castable wax resin. **H **3-D printed monoblock denture

**Fig. 2:**
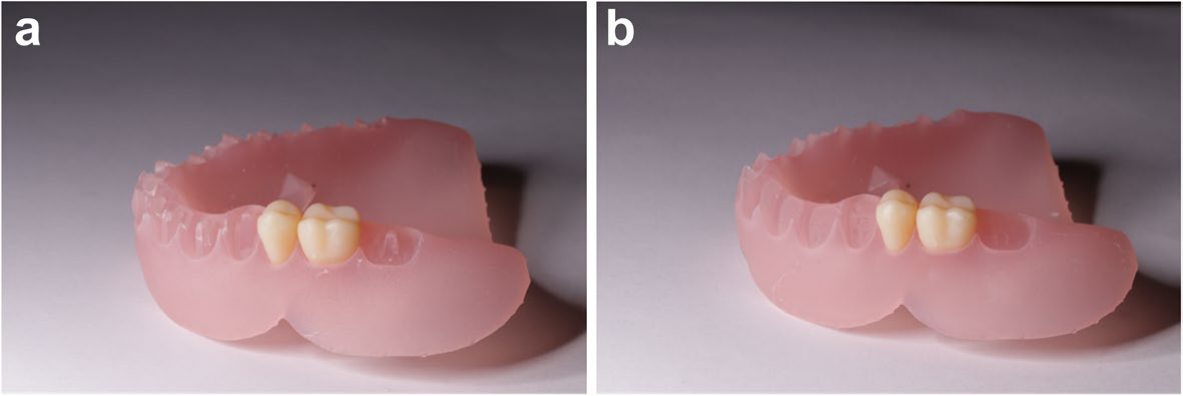
3D Printed denture bases. **A** Thimbled design. **B** Socketed design

Complete dentures were designed by using CAD software program (Exocad DentalCAD 3.0 Galway; Exocad GmbH). Teeth were also aligned in 3 forms: One-unit splinted design, 3-unit splinted design or unsplinted denture teeth. In addition to two designs for the teeth sockets of the denture base were employed: socketed design and thimble design. The dentures were divided into 8 groups (*n* = 10) according to the design used. Group I (unsplinted teeth/socketed design) (Fig. 1A), Group II (unsplinted teeth/thimbled design) (Fig. 1B), Group III (1-unit splinted teeth/socketed design) (Fig. 1C), Group IV (1-unit splinted teeth/thimble design) (Fig. 1D), Group V (3-unit splinted teeth/socketed design) (Fig. 1E), Group VI (3-unit splinted teeth/thimble design) (Fig. 1F), Group VII (Semi conventional pack and press) (Fig. 1G), Group VIII (Monoblock) (Fig. 1H). Groups from I -VI were 3D-printed by using SLA 3D-printer (Form 2; Formlabs Inc.) (Denture teeth A2, Formlabs) (Denture base LP, Formlabs). Group VII was 3D-printed by using castable wax resin (Castable wax, Formlabs Inc.) then flasked in a conventional manner. Group VIII was 3D printed by using SLA 3D-printer (Form 2; Formlabs Inc.) (Denture teeth A2, Formlabs). The thimble base groups were designed with cylindrical projections and a flat buccal surface to serve as an anti-rotational mean. Posterior sockets were designed with thimbles of 3 mm depth, 1.5 mm in width and 4 mm in length (Fig. 2). Anterior sockets were designed with thimbles of 4 mm depth, 1.5 mm in width and 4 mm in length. The respective denture teeth were designed with holes corresponding to those projections. Denture teeth were arranged by using the CAD software (Exocad 3.0 Galway; Exocad GmbH) far enough vertically not to interfere with the maxillary models, this allowed the preservation of the original basal tooth form of the denture teeth without the elimination of interferences. Denture teeth off-set was set to 0.2-mm for all groups [24].

### Three-dimensional printing and post-processing

For the groups with splinted teeth, the dimensions of the connectors were set to 9-mm^2^ [25]. Connectors were restricted to the proximal surfaces of the denture teeth. Three pyramids were designed and added on the palatal portion of polished surface by using CAD software program (Meshmixer; Autodesk Inc) to aid in superimposition. Supports were placed on the denture bases away from the teeth sockets. For the denture teeth supports were placed away from the basal and the occlusal surfaces (Preform, Fromlabs). Denture bases and denture teeth were printed with a 90 degrees orientation providing superior accuracy [26, 27]. Layer thickness was set to 50 µm for denture base resin (Denture base LP, Formlabs), denture teeth (Denture teeth A2, Formlabs) and castable wax resin (Castable wax, Formlabs Inc.). Dentures were then washed using isopropylalchol (IPA) in an ultrasonic bath for 90 s, then the supports were removed by using diamond discs, the denture was rinsed again using IPA for another 90 s. Groups from I-IV the denture teeth were bonded by using the following procedure: Denture base was seated on the master model, a few drops of uncured denture base resin were added by using a 3 cm^3^ syringe (Denture base LP, Formlabs) in the denture bases, teeth were placed in their respective positions, a splint was then secured in place, the assembly was then cured by using a light cure device (Bluephase N MC, Ivoclar Vivadent). Post-curing was followed according to the manufacturer’s instructions; dentures were submerged in a glass container filled with heated glycerin and cured 30 min for each surface.

### Semi-conventional pack and press group

**Fig. 3:**
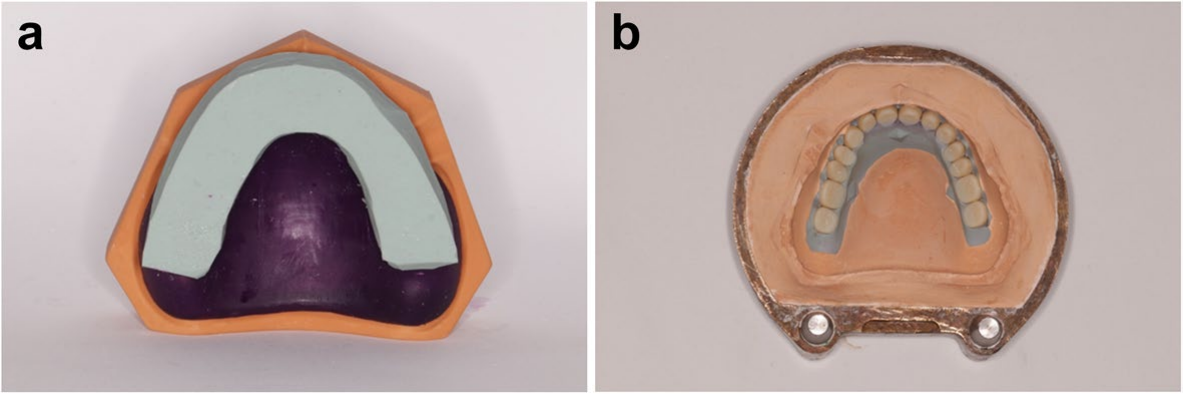
Semi-conventional pack and press processing; **A**: 3D printed castable resin warped with silicone around the denture teeth **B**: opened flask with the denture teeth in place

Regarding the semi-conventional denture (group VIII) a monoblock denture of the same design of the previous groups, was exported from Exocad the software and made hollow by using CAD software program (Meshmixer; Autodesk Inc) to allow softening of the castable wax during the washing step. The STL file was printed from a castable wax (Castable wax resin, Formlabs Inc). Silicone (Zetaplus, Zhermack) (Fig. 3). was placed around the teeth portion of the castable wax, to allow complete seating of the denture teeth in their respective positions. The castable wax denture was then flasked. After washing out, ready-made denture teeth (Myerson co) which are an exact copy of the printed teeth were set to their respective positions in the mold, acrylic resin is packed, and the denture is cured in a conventional manner. All dentures were then finished and prepared for scanning.

### Denture scanning, superimposition

**Fig. 4:**
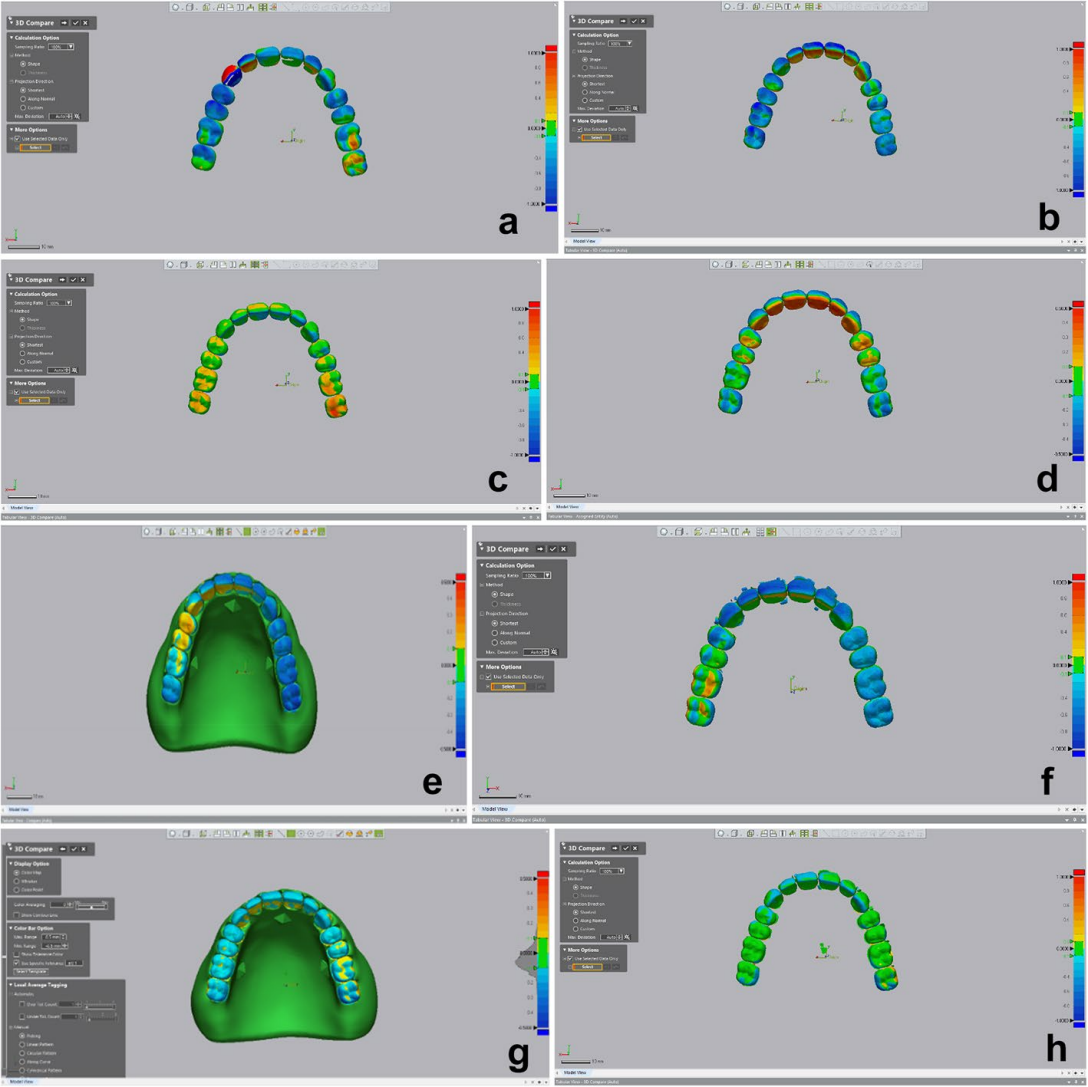
Different denture design groups. **A **Unsplinted socketed denture. **B **Unsplinted thimble denture. **C **One-unit splinted socketed denture. **D **One-unit splinted thimbled denture. **E **3-unit socketed denture. **F **3-unit splinted thimbled denture. **G **3-D printed monoblock denture. **H **Semi-conventional pack & press

The cameo and the fitting surface were scanned by using a desktop scanner (Medit T710, Medit Corp), the pre and post processing STL files were superimposed by using surface matching software (Geomagic® Control X, 3D systems) (Fig. 4). Superimposition was attained through initial best fit matching by finding points in common between the pre- and postprocessing files. The occlusal surface was excluded from the superimposition. Measurements were made at the 14 denture teeth for each of the 80 dentures. Deviations for each tooth were measured individually by using the 3D comparison function (Geomagic controlX). This approach will calculate a deviation value for every vertex in the measured data. Each tooth was selected as a separate region, the total number of points within each region in the 3D compare will be included. Deviations were set to have maximum critical values of ± 0.5 mm and a maximum nominal of ± 0.1 mm.

### Statistical evaluation

Each denture tooth was made as a separate region, then the 3D compare function was used for each tooth**.** Data was collected and statistically analysed with a statistical software program (IBM SPSS, version 23, Armonk). Normality was checked for all variables by using the Shapiro Wilk test and Q-Q plots. Differences in the RMS (Root Mean Square) estimates were assessed using the Kruskal Wallis test followed by Dunn’s post hoc test with Bonferroni adjustment. Reliability of the measurements was assessed using Intraclass Correlation Coefficient (ICC). All tests were two tailed and the significance of level was set at (*P* < 0.05).

## Results

In the present study the amount of deviation in teeth position was recorded in all groups. Sharpio-Wilko test revealed that the data were not normally distributed. Comparison between the 8 study groups was done by using Kruskal Wallis test. Deviation threshold values were set to a range of + 0.5 and − 0.5mm. Occlusal trueness was represented by the median of the RMS. Significance was set at *P* < 0.05. Regarding the RMS values, there was an excellent intra-rater agreement (ICC = 0.750, 95% CI: 0.719–0.777), with Minimal Detectable Change (MDC) of 0.035. The coefficient of repeatability is 0.07 (95% CI: 0.067–0.073).

### Table 1

#### Overall denture teeth

Table 1 presents the results of the 3D comparison where RMS values were used as they provide an absolute value in which positive values are not offset by negative values. The median and interquartile ranges of the root mean squared values were presented in all groups. The median of the overall RMS deviation values were significantly different among the groups, the lowest deviation was reported group VII: Monoblock (0.104), followed by Group III: socketed base/1 unit splinted teeth (0.125), Group V: socketed base/3-unit splinted design (0.152), Group VIII: semi-conventional pack and press (0.163), Group VI: thimble base/3-unit splinted teeth (0.170), group I socketed base/unsplinted teeth (0.184), group IV: thimble base/1 unit splinted teeth (0.202), group II: thimble base/unsplinted teeth (0.216). After a pair wise comparison a non-statistically significant difference was found among groups I and II; groups IV, I and II; groups VI, I and IV; group VIII,I,V,VI.

**Table 1:** Comparison of the RMS deviation values (mm) resultant from the 3D comparison of different denture teeth groups

	**All Root Mean Square (mm)**	**Groups**							
		Separate Socket (*n* = 140) (a)	Separate Thimble (*n* = 140) (b)	Connected Socket (*n* = 140) (c)	Connected Thimble (*n* = 140) (d)	Bridges Socket (*n* = 140) (e)	Bridges Thimble (*n* = 140) (f)	Semi-Conventional Pack and Press (*n* = 140) (g)	Monoblock (*n* = 140) (h)
**Overall denture teeth**	- Min. – Max - Median - 95% CI of the median - 25 th − 75th Percentile	0.064–0.470 0.184^a,b,d,f,g^ 0.174–0.194 0.151–0.226	0.087–0.595 0.216^a,b,d^ 0.204–0.223 0.167–0.254	0.067–0.262 0.125^c^ 0.118–0.133 0.103–0.147	0.065–0.419 0.202^a,b,d^ 0.188–0.225 0.149–0.278	0.051–0.285 0.152^e,f,g^ 0.142–0.164 0.119–0.181	0.052–0.363 0.170^a,e,f,g^ 0.157–0.182 0.131–0.203	0.083–0.292 0.163^a,e,f,g^ 0.155–0.175 0.131–0.211	0.060–0.283 0.104^ h^ 0.100–0.112 0.087–0.125
	**Test of significance** ***p*-value**	H_(df=7)_ = 66.807 *p* <.001*							
**All Anterior denture teeth**	- Min. – Max - Median - 95% CI of the median - 25 th − 75th Percentile	0.087–0.304 0.176^a,f,g^ 0.166–0.185 0.151–0.198	0.126–0.511 0.238^b,d,f^ 0.219–0.263 0.199–0.306	0.067–0.204 0.119^c,e,g,h^ 0.107–0.126 0.103–0.136	0.170–0.419 0.282^b,d^ 0.271–0.305 0.249–0.321	0.051–0.224 0.149^c,e,g^ 0.133–0.160 0.121–0.172	0.070–0.363 0.191^a,b,f^ 0.182–0.210 0.163–0.242	0.088–0.216 0.149^a,c,e.g^ 0.137–0.160 0.118–0.175	0.063–0.226 0.104^c,h^ 0.098-.116 0.089–0.128
	**Test of significance** ***p*-value**	H_(df=7)_ = 293.793 *p* <.001*							
**Posterior Denture teeth**	- Min. – Max - Mean ± SD - Median - 95% CI of the median - 25 th − 75th Percentile	0.064–0.470 0.204 ± 0.080 0.196^a,b,g^ 0.177–0.220 0.150–0.244	0.087–0.595 0.198 ± 0.077 0.200^a,b,g^ 0.177–0.217 0.145–0.229	0.075–0.262 0.141 ± 0.047 0.135^c,d,e,f^ 0.122–0.140 0.105–0.164	0.065–0.297 0.1610 ± 0.050 0.166^c,d,e,f,g^ 0.142–0.180 0.120–0.190	0.071–0.285 0.161 ± 0.050 0.156^c,d,e,f,g^ 0.146–0.176 0.119–0.196	0.052–0.228 0.151 ± 0.039 0.149^c,d,e,f^ 0.135–0.166 0.117–0.187	0.083–0.292 0.188 ± 0.054 0.187^a,b,d,e,g^ 0.164–0.218 0.143–0.229	0.060–0.283 0.1090 ± 0.039 0.104^ h^ 0.095–0.112 0.084–0.124
	**Test of significance** ***p*-value**	H_(df=7)_ = 152.190 *p* <.001*							

Significance value have been adjusted by the Bonferroni correction for multiple tests

*Different Superscript Letters denote statistical significance*

*n* Number of denture teeth, *Min–Max* Minimum – Maximum, *SD* Standard Deviation, *CI* Confidence interval, *H* Kruskal–Wallis H,

^*^Statistically significant (*p* <.05) a separate socket, b separate thimble, c connected socket, d connected thimble, e bridges socket, f bridges thimble, f bridges thimble, g semi conventional, h monoblock

#### Anterior denture teeth

Regarding the anterior teeth, the median and interquartile range values (mm) measured in all groups were presented in Table 2. Deviation values were significantly different among the groups, with the lowest deviation was reported in group VII: Monoblock (0.104) mm, followed by group III socketed base/1 unit splinted teeth (0.119), group V: socketed base/3-unit splinted teeth (0.149), group VIII: semi-conventional pack and press (0.149), group I: socketed base/unsplinted teeth (0.176), group VI: thimble base/3 unit splinted teeth (0.191), group II: thimble base/unsplinted teeth (0.238), group IV: thimble base/1-unit splinted teeth (0.282).

**Table 2:** Comparison of the RMS deviation values (mm) resultant from the 3D comparison of different denture teeth types

	**All Root Mean Square (mm)**	**Groups**															
		Separate Socket (*n* = 140)		Separate Thimble (*n* = 140)		Connected Socket (*n* = 140)		Connected Thimble (*n* = 140)		Bridges Socket (*n* = 140)		Bridges Thimble (*n* = 140)		Semi-Conventional Pack and Press (*n* = 140)			Monoblock (*n* = 140)
**Incisors**	- Min. – Max - Median - 95% CI of the median - 25 th − 75th Percentile	0.125–0.304 0.178^a,e,f,g^ 0.164–0.195 0.153–0.205		0.134–0.511 0.262 ^b,d,f^ 0.225–0.301 0.210–0.323		0.068–0.183 0.119 ^c,e g h^ 0.107–0.129 0.105–0.136		0.198–0.419 0.308^b,d^ 0.295–0.322 0.276–0.344		0.051–0.224 0.156^a,c,e,g^ 0.138–0.169 0.130–0.176		0.086–0.345 0.198^a,b,f^ 0.176–0.221 0.167–0.244		0.088–0.216 0.154^a,c,e,g^ 0.129–0.177 0.117–0.186			0.063–0.185 0.106^c,h^ 0.096–0.120 0.089–0.125
	**Test of significance** ***p*-value**	H_(df=7)_ = 209.578 *p* <.001*															
**Canines**	- Min. – Max - Median - 95% CI of the median - 25 th − 75th Percentile	0.087–0.282 0.171^a,b,c,e,f,g^ 0.139–0.184 0.138–0.184		0.126–0.433 0.219^a,b,d f^ 0.189–0.246 0.182–0.250		0.067–0.204 0.113^a,c,e,g,h^ 0.097–0.134 0.096–0.135		0.170–0.329 0.252^b,d,f^ 0.227–0.271 0.225–0.276		0.056–0.212 0.126^a,c,e,g,h^ 0.095–0.150 0.094–0.154		0.070–0.363 0.185^a,b,d,f^ 0.165–0.229 0.162–0.235		0.098–0.186 0.139^a,c,e,g,h^ 0.135–0.159 0.125–0.159			0.077–0.226 0.101^c,e,g,h^ 0.090–0.130 0.088–0.130
	**Test of significance** ***p*-value**	H_(df=7)_ = 89.606 *p* <.001*															
**Premolars**	- Min. – Max - Median - 95% CI of the median - 25 th − 75th Percentile	0.064–0.470 0.213^a,b,d,e,g^ 0.186–0.242 0.159–0.255		0.091–0.595 0.208^b,d,e,g^ 0.178–0.221 0.160–0.230		0.075–0.258 0.129^c,d,e,f,h^ 0.109–0.139 0.100–0.144		0.088–0.297 0.178^a,b,c,d,e,f,g^ 0.145–0.189 0.134–0.195		0.082–0.285 0.156^a,b,c,d,e,f,g^ 0.132–0.181 0.114–0.192		0.091–0.228 0.145^c,d,e,f,g^ 0.125–0.169 0.114–0.188		0.090–0.280 0.184^a,b,d,e,f,g^ 0.159–0.218 0.143–0.227		0.066–0.283 0.105^c,h^ 0.091–0.111 0.085–0.119	
	**Test of significance** ***p*-value**	H_(df=7)_ = 89.138 *p* <.001*															
**Molars**	- Min. – Max - Median - 95% CI of the median - 25 th − 75th Percentile	0.067–0.456 0.184^a,b,d,e,f,g^ 0.152–0.222 0.149–0.233	0.087–0.324 0.185^a,b,c,d,e,f,g^ 0.145–0.217 0.143–0.226		0.078–0.262 0.139^b,c,d,e,f^ 0.118–0.164 0.107–0.182		0.065–0.283 0.149^a,b,c,d,e,f,g^ 0.120–0.174 0.106–0.187		0.071–0.273 0.156^a,b,c,d,e,f,g^ 0.125–0.192 0.119 ± 0.201		0.052–0.224 0.150^a,b,c,d,e,f,g^ 0.130–0.179 0.121–0.184		0.083–0.292 0.187^a,b,d,e,f,g^ 0.162–0.225 0.144–0.231		0.060–0.230 0.104^ h^ 0.090–0.119 0.077–0.126		
	**Test of significance** ***p*-value**	H_(df=7)_ = 66.807 *p* <.001*															

Significance value have been adjusted by the Bonferoni correction for multiple tests

*n* Number of denture teeth, *Min–Max* Minimum – Maximum, *SD* Standard Deviation, *CI* Confidence interval, *H* Kruskal–Wallis H

^*^Statistically significant (*p* <.05) a separate socket, b separate thimble, c connected socket, d connected thimble, e bridges socket, f bridges thimble, f bridges thimble, g semi conventional, h monoblock

#### Posterior denture teeth

As for the posterior teeth, the least deviation was reported in group VII: Monoblock (0.104), followed by group III: socketed base/1 unit splinted teeth (0.135), group VI: thimble base/3-unit splinted teeth (0.149), group V:socketed base/3-unit splinted teeth (0.156), group IV: thimble base/1-unit splinted teeth (0.166), group VIII: semi-conventional pack and press (0.187), group I: socketed base/unsplinted teeth (0.196), group II: thimble base/unsplinted teeth (0.200). The posterior teeth showed generally higher deviation values than the anterior teeth except for groups II, IV, VI and VII. When average deviation values for the 3D comparison were analysed, negative values of teeth deviations were noted across all groups except for group VIII: semi-conventional pack and press.

### Table 3

Regarding the teeth categories, 3D comparison was conducted, and RMS deviation of the incisors, canines, premolars and molars were statically compared. With respect to the incisors, Group VII: Monoblock dentures had the least median value of (0.106) followed by group III: socketed base/1-unit splinted teeth (0.119), group VIII: semi-conventional pack and press (0.154), group V: socketed base/3-unit splinted teeth (0.156), group I: separate socket (0.178), group VI: thimble base/3-unit splinted teeth (0.198), group II: thimble base/unsplinted teeth (0.262) and the highest deviation was reported in group IV: thimbled base/1-unit splinted teeth (0.308). A higher deviation in the incisors than the canines was reported across all groups except for group V and group VI. The deviation was variable when comparing the premolars with the molars. Some groups demonstrated a greater deviation across the premolars namely groups I,II,IV and VII. Group V demonstrated an equal amount of deviation in both premolars and molars. Groups III, VI and VIII demonstrated a smaller amount of deviation as compared to the molars (Table 3).

**Table 3:** Comparison of the RMS deviation values (mm) resultant from the 3D comparison of different denture teeth

Central	Root Mean Square (mm)	Groups							
		Separate Socket (*n* = 20)	Separate Thimble (*n* = 20)	Connected Socket (*n* = 20)	Connected Thimble (*n* = 20)	Bridges Socket (*n* = 20)	Bridges Thimble (*n* = 20)	Semi-Conventional Pack and Press (*n* = 20)	Monoblock (*n* = 20)
	- Median - 25 th − 75th Percentile	0.125—0.257 0.162^a,c,e,f,g^ 0.146—0.194 0.145—0.195	0.140–0.511 0.323^b,d,f,h^ 0.225–0.372 0.220–0.380	0.068–0.183 0.114^a,c,e,g,h^ 0.104–0.133 0.103–0.136	0.216–0.419 0.316^b,d,f^ 0.295–0.346 0.286–0.351	0.063–0.224 0.162^a,c,e,f,g,h^ 0.133–0.177 0.130–0.178	0.122–0.345 0.219^a,b,d,e,f,g^ 0.176–0.267 0.176–0.274	0.099–0.200 0.152^a,c,e,f.g,h^ 0.120–0.165 0.118–0.168	0.063–0.136 0.109^c,e,g,h^ 0.095–0.123 0.093–0.124
	**Test of significance** ***p*-value**	H_(df=7)_ = 112.449 *p* <.001*							
**Laterals**	- Min. – Max - Median - 95% CI of the median - 25 th − 75th Percentile	0.151–0.304 0.193^a,b,e,f,g^ 0.174–0.208 0.171–0.210	0.134–0.312 0.237^a,b,d,f^ 0.204–0.270 0.199–0.276	0.087–0.180 0.124^c,e,g,h^ 0.110–0.135 0.109–0.136	0.198–0.391 0.302^b,d^ 0.249–0.317 0.245–0.319	0.051–0.217 0.154^a,c,e,f,g,h^ 0.132–0.172 0.130–0.172	0.086–0.312 0.186^a,b,e,f^ 0.161–0.210 0.160–0.212	0.088–0.216 0.179^a,c,e,f,g,h^ 0.117–0.198 0.109–0.199	0.076–0.185 0.102^c,e,g,h^ 0.087–0.132 0.087–0.132
	**Test of significance** ***p*-value**	H_(df=7)_ = 99.826 *p* <.001*							
**Canines**	**Canines Root Mean Square (mm**^**2**^**)**								
		Separate Socket (*n* = 20)	Separate Thimble (*n* = 20)	Connected Socket (*n* = 20)	Connected Thimble (*n* = 20)	Bridges Socket (*n* = 20)	Bridges Thimble (*n* = 20)	Semi-Conventional Pack and Press (*n* = 20)	Monoblock (*n* = 20)
	- Min. – Max - Median - 95% CI of the median - 25 th − 75th Percentile	0.087–0.282 0.171^a,b,c,e,f,g^ 0.139–0.184 0.138–0.184	0.126–0.433 0.219^a,b,d f^ 0.189–0.246 0.182–0.250	0.067–0.204 0.113^a,c,e,g,h^ 0.097–0.134 0.096–0.135	0.170–0.329 0.252^b,d,f^ 0.227–0.271 0.225–0.276	0.056–0.212 0.126^a,c,e,g,h^ 0.095–0.150 0.094–0.154	0.070–0.363 0.185^a,b,d,f^ 0.165–0.229 0.162–0.235	0.098–0.186 0.139^a,c,e,g,h^ 0.135–0.159 0.125–0.159	0.077–0.226 0.101^c,e,g,h^ 0.090–0.130 0.088–0.130
	**Test of significance** ***p*-value**	H_(df=7)_ = 89.606 *p* <.001*							
**First Premolar**	**Root Mean Square (mm)**	**Groups**							
		Separate Socket (*n* = 20)	Separate Thimble (*n* = 20)	Connected Socket (*n* = 20)	Connected Thimble (*n* = 20)	Bridges Socket (*n* = 20)	Bridges Thimble (*n* = 20)	Semi-Conventional Pack and Press (*n* = 20)	Monoblock (*n* = 20)
	- Min. – Max - Median - 95% CI of the median - 25 th − 75th Percentile	0.084–0.470 0.201^a,b,d,e,f,g^ 0.169–0.248 0.167–0.255	0.091–0.595 0.217^a,b,d,e,f,g^ 0.183–0.231 0.182–0.231	0.079–0.251 0.121^c,d,e,f,h^ 0.099–0.142 0.098–0.144	0.093–0.297 0.189 ^c,d,e,f,h^ 0.171–0.211 0.159–0.211	0.091–0.243 0.160^a,b,c,d,e,f,g,h^ 0.127–0.192 0.120–0.192	0.091–0.228 0.160^a,b,c,d,e,f,g,h^ 0.133–0.191 0.125–0.192	0.105–0.280 0.184^a,b,d,e,f,g^ 0.156–0.221 0.148–0.229	0.066–0.283 0.099 ^c,e,f,h^ 0.084–0.111 0.084–0.111
	**Test of significance** ***p*-value**	H_(df=7)_ = 49.646 *p* <.001*							
		**Pairwise Comparison using Dunn-Sidak Method**							
**Second Premolar**	**Root Mean Square (mm)**	**Groups**							
		Separate Socket (*n* = 20)	Separate Thimble (*n* = 20)	Connected Socket (*n* = 20)	Connected Thimble (*n* = 20)	Bridges Socket (*n* = 20)	Bridges Thimble (*n* = 20)	Semi-Conventional Pack and Press (*n* = 20)	Monoblock (*n* = 20)
	- Min. – Max - Median - 95% CI of the median - 25 th − 75th Percentile	0.064—0.354 0.221^a,b,d,e,f,g^ 0.154–0.245 0.148–0.261	0.099–0.376 0.180^a,b,c,d,e,f,g^ 0.137–0.219 0.136–0.220	0.075–0.258 0.136^b,c,d,e,f,g,h^ 0.103–0.141 0.102–0.143	0.088–0.267 0.148^a,b,c,d,e,f,g,h^ 0.125–0.180 0.119–0.181	0.082–0.285 0.153^a,b,c,d,e,f,g^ 0.114–0.186 0.113–0.189	0.103–0.208 0.132^a,b,c,d,e,f,g,h^ 0.111–0.169 0.110–0.170	0.090–0.280 0.179^a,b,c,d,e,f,g^ 0.145–0.226 0.143–0.227	0.068–0.176 0.106^c,d,f,h^ 0.090–0.124 0.088–0.125
	**Test of significance** ***p*-value**	H_(df=7)_ = 41.566 *p* <.001*							
**First Molar**	**First molar Root Mean Square (mm)**	**Groups**							
		Separate Socket (*n* = 20)	Separate Thimble (*n* = 20)	Connected Socket (*n* = 20)	Connected Thimble (*n* = 20)	Bridges Socket (*n* = 20)	Bridges Thimble (*n* = 20)	Semi-Conventional Pack and Press (*n* = 20)	Monoblock (*n* = 20)
	- Min. – Max - Median - 95% CI of the median - 25 th − 75th Percentile	0.067–0.456 0.186^a,b,c,e,f,g^ 0.150–0.229 0.148–0.231	0.087–0.324 0.196^a,b,c,e,f,g^ 0.145–0.230 0.145–0.231	0.078–0.262 0.130^a,b,c,d,e,f,g,h^ 0.105–0.164 0.105–0.174	0.065–0.283 0.113^c,d,e,f,h^ 0.096–0.141 0.094–0.148	0.071–0.239 0.150^a,b,c,d,e,f,g,h^ 0.115–0.179 0.111–0.189	0.052–0.204 0.135^a,b,c,d,e,f,g,h^ 0.121–0.157 0.117–0.168	0.085–0.292 0.176^a,b,c,e,f,g^ 0.142–0.225 0.140–0.227	0.060–0.193 0.094^c,d,e,f,h^ 0.075–0.117 0.074–0.117
	**Test of significance** ***p*-value**	H_(df=7)_ = 46.438 *p* <.001*							
**Second Molar**	**Root Mean Square (mm)**	**Groups**							
		Separate Socket (*n* = 20)	Separate Thimble (*n* = 20)	Connected Socket (*n* = 20)	Connected Thimble (*n* = 20)	Bridges Socket (*n* = 20)	Bridges Thimble (*n* = 20)	Semi-Conventional Pack and Press (*n* = 20)	Monoblock (*n* = 20)
	- Min. – Max - Median - 95% CI of the median - 25 th − 75th Percentile	0.080–0.364 0.174^a,b,c,d,e,f,g^ 0.149–0.233 0.149–0.234	0.096–0.315 0.177^a,b,c,d,e,f,g^ 0.136–0.217 0.129–0.219	0.082–0.259 0.151^a,b,c,d,e,f,g,h^ 0.119–0.179 0.118–0.185	0.107–0.277 0.182^a,b,c,d,e,f,g^ 0.166–0.193 0.154–0.196	0.107–0.273 0.175^a,b,c,d,e,f,g^ 0.146–0.205 0.135–0.206	0.105–0.224 0.166^a,b,c,d,e,f,g,h^ 0.139–0.189 0.133–0.195	0.083–0.271 0.205^a,b,c,d,e,f,g^ 0.162–0.242 0.159–0.242	0.065–0.230 0.118^c,f,h^ 0.095–0.132 0.094–0.135
	**Test of significance** ***p*-value**	H_(df=7)_ = 30.541 *p* <.001*							

Significance value have been adjusted by the Bonferoni correction for multiple tests

*N* Number of denture teeth, *Min–Max* Minimum – Maximum, *SD* Standard Deviation, *CI* Confidence interval, *H* Kruskal–Wallis H,

^*^Statistically significant (*p* <.05) a separate socket, b separate thimble, c connected socket, d connected thimble, e bridges socket, f bridges thimble, f bridges thimble, g semi conventional, h monoblock

### Colour maps

Colour maps illustrate directional deviation among various groups. A green colour indicates minimal deviation, a shift towards the red colour indicates a deviation away from the intaglio surface of the denture and a shift towards the blue colour indicates a deviation towards the intaglio surface of the denture. Groups I, II and IV demonstrate a blue colour range among most teeth except for the palatal surfaces of the anterior teeth that demonstrate a shift towards the yellow to red colours. Group III demonstrates a variation of the green and yellow colours; The yellow colour was observed in the posterior teeth area. Groups V, VI, VII and VIII exhibited a range of blue and yellow colours. The yellow colour was noticed mainly among the posterior teeth and the palatal surface of the anterior teeth.

## Discussion

A significant difference in the teeth deviations across the 8 study groups examined in this study was detected. Consequently, the null hypothesis was rejected. Mean deviations were reported to be in the range of (0.104–0.282) mm making them within the acceptable range of occlusal deviation reported by Kanazawa et al. [23].

Occlusal errors in 3D-printed dentures could occur either during the printing of the denture base and the denture teeth or during the processing of printed dentures or during the bonding procedure of denture teeth to the denture base. Denture processing was done by the same dentist across the whole study to avoid any errors that could be caused by different manipulation. ICC test results denoted excellent reliability. For the groups with splinted teeth, the dimensions of the connectors were set to 9-mm^2^providing enough strength yet not interfering with the esthetics [25].

This study aimed to reduce occlusal errors that occur during bonding procedures, therefore different teeth assemblies and anti-rotational features (thimbles) were used. During the bonding procedure an occlusal splint was utilised to standardize the amount and direction of pressure exerted on the denture teeth. Moreover, a high accuracy laboratory scanner (Medit T710, Medit Corp) that could generate reference scans was used [23]. In order to eliminate the effect of any occlusal error on the results, superimposition was attained excluding the occlusal surface and 3 pyramids were added on the polished surface, thereby increasing the area and the planes of superimposition. Deviations for each tooth were measured individually by using the 3D comparison function (Geomagic controlX) this approach will calculate a deviation value for every vertex in the measured data. Each tooth was selected as a separate region, the total number of points within each region in the 3D compare will be included. This function considers the movement of each tooth from all aspects, providing a more precise depiction than by using single tooth points. In contrast to Goodacre et al [8] and Thabet el al [12] in which superimposition was done by using the entire denture surface including the surfaces to be measured. Furthermore, single tooth points were used in the aforementioned studies to measure the deviation.

In the present study the median deviation of the overall RMS value in the Semi-conventional pack and press group was reported to be 0.163 mm and the IQR (0.131–0.211) mm. In contrast to Goodacre et al., [8] where a median deviation distance of 0.125 mm and the IQR (− 0.10–0.375) mm in the conventional pack and press group was reported. Differences in those values were expected and are attributed to the different measurements used, namely the RMS and average deviation distance. Moreover, the current study used a 3D printed monolithic denture to serve as a wax pattern with a layer of silicone around the teeth, this might account for the smaller interquartile range reported. Furthermore, the aforementioned study did not clarify whether the denture teeth sets were splinted or separate in the CAD/CAM bonded group, which might account for the differences observed. In addition, the CAD/CAM monolithic denture was milled in contrast to the present study where the monoblock denture was printed. However, Goodacre et al., have reported that the highest occlusal accuracy was recorded in monolithic CAD/CAM denture which is in agreement with the present study.

RMS deviation values for the SLA printed maxillary denture teeth reported by Deng et al. [6] were smaller yet close to those reported in the present study. Those differences could be attributed to the different resin materials used, the printing angle which was set to 30° in the previously mentioned study in contrast to 90° in the present study. Also, the layer thickness was set to 100µm by Deng et al. and 50µm in the current study.

Mean value measurements in the present study conflicted with the study conducted by Thabet Y et al. [12]. Those differences were expected; possibly due to the different type of resin used. In addition, a different method was used for the measurement of the occlusal trueness where only 4 teeth per denture were used to calculate the deviation and 2 points on the cusp tips of the molars and the canines were utilised. Moreover, the printing angle was not stated in the aforementioned study, differences in the printing angle could account to the different occlusal deviation values obtained. In the present study, RMS values for average teeth deviations were utilized for statistical analysis to eliminate the offset caused by negative and positive values, consequently providing a more accurate representation of tooth deviations [28]. Studies reporting mean deviations have smaller values as compared to the present study and those reporting RMS values because of the offset caused by negative values [8, 12].

Colour maps demonstrated negative deviation of printed dentures, which aligned with the findings of You et al., regarding CAD-CAM groups [29]. That could be attributed to the centripetal shrinkage of the photopolymer resin. The thimbled basal tooth forms could have increased the effect of the centripetal shrinkage, resulting in the increased negative deviations seen on the labial surface of the anterior teeth and the increased positive deviations illustrated on the palatal surface of the anterior teeth in groups II, IV and VI as compared to groups I,III and V. Although the unsplinted denture teeth and the 3-unit splinted denture teeth were expected to have a higher occlusal trueness, due to the reduction of the sagging effect away from the printing platform while printing the denture teeth. However, 1-unit splinted socket denture design demonstrated an improved overall occlusal trueness. This could be attributed to the splinting effect, which helps reduce the occlusal errors during the bonding procedure of the denture base to the denture teeth.

The thimble denture base design was expected to improve the occlusal trueness. However, this proved untrue. It is likely that the film thickness of the uncured photopolymer resin as well as the thimbles did not allow the precise positioning of the denture teeth in their respective positions. Moreover, it has been noted that higher deviation across the incisors than the molars was noted in the thimbled basal design groups. This is likely to be attributed to the longer thimbles in the anterior region that hindered the complete seating of the incisors.

Limitations of this study is the inclusion of printed maxillary dentures only. Further investigations should include milled maxillary and mandibular dentures. As well as the effect on the vertical dimension of occlusion clinically. Occlusal trueness was assessed under dry conditions. Additionally, the layer thickness was set to 50 µm, because of the software used (Preform; Formlabs) does not provide the 100 µm layer thickness as an option for the materials used. It has been reported that a 100 µm layer thickness provides superior accuracy as compared to the 50 µm layer thickness [29]. Furthermore, the effect of the thimbles on the tooth-denture base bond has not been investigated, subsequent studies should examine the effect of incorporating thimbles on the bond between the denture base and the teeth as well as investigate the occlusal trueness of the different splinting assemblies and basal designs after cyclic loading, body temperature and in artificial saliva. Future studies should also aim to increase sample sizes to enhance the reliability of findings.

## Conclusions

Based on the findings of this in vitro study, the following were concluded:

1. Monoblock design has provided significantly the highest occlusal trueness among 3D-printed dentures groups
2. One-unit splinted design provided the highest occlusal trueness among the teeth bonded CAD-CAM dentures.
3. Splinting denture teeth has a superior effect on the occlusal trueness than incorporating an anti-rotational means as thimbles
4. Semi-conventional pack and press yielded a comparable occlusal trueness to CAD-CAM bonded 3D printed dentures.

## Data Availability

The raw data of the present study is available at: https://figshare.com/articles/dataset/3D_Printed_Dentures_with_different_socket_forms/27297150?file=49973355.
